# Infarto medular secundario a tratamiento endovascular de aneurisma de aorta toraco-abdominal : Reporte de un caso

**DOI:** 10.31053/1853.0605.v81.n1.41439

**Published:** 2024-03-27

**Authors:** Luis Alfredo Hernández Villarroel, Mercedes de Lera Alfonso

**Affiliations:** 1 Hospital Clínico Universitario de Valladolid Valladolid

**Keywords:** médula espinal, enfermedades de la médula espinal, isquemia de la médula espinal, paraparesia, aneurisma de la aorta, spinal cord, spinal cord diseases, spinal cord ischemia, paraparesis, aortic aneurysm, médula espinal, doenças da medula espinal, isquemia do cordáo espinal, paraparesia, aneurisma aórtico

## Abstract

**Introducción:**

El infarto medular es una patología severa e infrecuente, que representa el 1% del total de ictus isquémicos, siendo además una complicación rara de distintos procedimientos quirúrgicos. Es causado por la interrupción aguda del flujo sanguíneo de la médula espinal, manifestándose con déficits neurológicos clínicos relacionados con el territorio vascular afectado.

**Métodos:**

Presentamos el caso de un paciente de 80 años, con factores de riesgo cardiovascular, quien presenta en día postquirúrgico 13, tras colocación de endoprótesis vascular por aneurisma toraco-abdominal aparición brusca de paraparesia con progresión a paraplejía e hipoestesia en ambas extremidades inferiores. Angio-TC de aorta descarta complicación local en la endoprótesis. RM medular mostró imágenes compatibles con Infarto agudo de médula dorsal desde el nivel D9. El paciente no fue subsidiario de tratamiento revascularizador. El tratamiento consistió en medidas de soporte.

**Resultados:**

Al alta el paciente presentaba paraplejia e hipoestesia de ambas extremidades inferiores con incontinencia fecal y urinaria.

**Conclusión:**

El infarto de la médula espinal puede estar limitado a un territorio vascular o estar más extendido según su patogenia. La afectación de la arteria espinal anterior es la más común y se caracteriza por déficits motores bilaterales y pérdida de la sensibilidad termoalgésica, pudiendo llegar a producir un gran impacto en la calidad de vida de los pacientes. Su etiología es variada, incluyéndose la cirugía aórtica dentro de sus causas. La RM es muy útil para su diagnóstico y actualmente no existen guías clínicas para el tratamiento óptimo.

## Abstract (en)

**Introduction:**

Medullary infarction is a severe and infrequent pathology, which represents 1% of all ischemic strokes, and is also a rare complication of different surgical procedures. It is caused by the acute interruption of the blood flow of the spinal cord, manifesting itself with clinical neurological deficits related to the affected vascular territory.

**Methods:**

We present the case of an 80-year-old patient, with cardiovascular risk factors, who is present on post-surgical day 13, after placement of a vascular endoprosthesis for a thoracoabdominal aneurysm, sudden appearance of paraparesis with progression to paraplegia and hypoesthesia in both lower extremities. CT angiography of the aorta rules out local complications in the endoprosthesis. Medullary MRI showed images compatible with acute dorsal medullary infarction from level D9.

**Results:**

On discharge, the patient presented paraplegia and hypoesthesia of both lower extremities with fecal and urinary incontinence.

**Conclusión:**

Spinal cord infarction may be limited to a vascular territory or be more extensive according to its pathogenesis. The affectation of the anterior spinal artery is the most common and is characterized by bilateral motor deficits and loss of thermoalgesic sensitivity, which could have a great impact on the quality of life of patients. Its etiology is varied, including aortic surgery within its causes. MRI is very useful for its diagnosis and currently there are no clinical guides for the optimal treatment.

## Resumo

**Introdução:**

O infarto medular é uma patologia grave e infrecuente, que representa el 1% do total de ictus isquêmicos, sendo também uma complicação rara de diferentes procedimentos cirúrgicos. É causado pela interrupção aguda do fluxo sanguíneo da medula espinhal, manifestando-se com déficits neurológicos clínicos relacionados ao território vascular afetado.

**Caso clínico:**

Apresentamos o caso de um paciente de 80 anos, com fatores de risco cardiovascular, que se apresentou no dia 13 pós-cirúrgico, tras colocación de endoprótese vascular por aneurisma toraco-abdominal aparição brusca de paraparesia com progressão a paraplejía e hipoestesia em ambas as extremidades inferiores. Angio-TC de aorta descarta complicações locais na endoprótese. RM medular mostró imagens compatíveis com Infarto agudo de médula dorsal desde o nível D9.

**Resultados:**

Na alta, o paciente apresentou paraplegia e hipoestesia de ambas as extremidades inferiores com incontinência fecal e urinária.

**Conclusão:**

O infarto da medula espinal pode estar limitado a um território vascular ou estar mais estendido de acordo com a patogenia. A afetação da artéria espinal anterior é a mais comum e se caracteriza por déficits motores bilaterais e perda da sensibilidade termoalgésica, podendo levar a produzir um grande impacto na qualidade de vida dos pacientes. Sua etiologia é variável, incluindo a cirurgia aórtica dentro de suas causas. La RM é muito útil para seu diagnóstico e atualmente não existem guias clínicos para o tratamento ideal.

**CONCEPTOS CLAVE**

**Qué se sabe sobre el tema.**

El infarto medular es una patología infrecuente que representa el 1% del total de ictus isquémicos. Es causado por la interrupción aguda del flujo sanguíneo de la médula espinal resultando en isquemia, infarto y disfunción aguda de la médula espinal con déficits neurológicos clínicos relacionados con el territorio vascular afectado. Siendo, una complicación rara de distintos procedimientos quirúrgicos, como la cirugía aórtica y de columna.

**Qué aporta este trabajo.**

Existen pocos casos descritos de infarto medular secundario a tratamiento endovascular de aneurisma aórtico, por lo que este articulo podría contribuir en la discusión de esta patología de baja frecuencia, cuyas manifestaciones clínicas aparecen de forma brusca y la exploración neurológica es muy importante en el período postoperatorio debiendo mantener un alto grado de sospecha para identificar causas tratables.

**Divulgación**

El infarto medular es una patología infrecuente producida por interrupción del flujo sanguíneo de la médula espinal provocando la alteración de la función medular según el segmento afectado, pudiendo clínicamente presentar disminución o ausencia de la fuerza muscular de las extremidades inferiores. Sus causas son variadas, pero puede estar asociada a intervenciones quirúrgicas, incluyendo la reparación de aneurismas aórticos toracoabdominales.

## Introducción

El infarto medular es infrecuente y representa el 1% del total de ictus isquémicos. Su diagnóstico constituye un reto debido a que los signos clínicos con los que suele presentarse tienen poca especificidad y gran variabilidad^(1)^. Es causado por la interrupción aguda del flujo sanguíneo de la médula espinal resultando en isquemia, infarto y disfunción aguda de la médula espinal con déficits neurológicos clínicos relacionados con el territorio vascular afectado^(2)^, cuya máxima gravedad puede ser alcanzada dentro de la primeras 12 horas en el 50% de los pacientes y puede variar desde paraplejía hasta paresia mínima, siendo frecuentemente acompañada por dorsalgia en hasta un 70% de los pacientes, típicamente a nivel de la lesión^(3)^.

La isquemia iatrogénica de la médula espinal es una complicación rara y debilitante de distintos procedimientos quirúrgicos, como la cirugía aórtica y de columna, incluyendo la reparación de aneurismas aórticos toracoabdominales, que precipitan diversos grados de lesión permanente^(4)^, probablemente debido a que el pinzamiento de la aorta podría empeorar el flujo espinal^(5)^. Algunos estudios han indicado que el nivel más habitual de lesión iatrogénica se halla en las regiones cervical y torácica baja^(4)^. Han sido pocos los casos reportados en la literatura sobre isquemia medular provocada por la cirugía, ya sea abierta o por métodos endovasculares^(5)^.

## Caso Clínico

Paciente hombre de 80 años, sin alergias conocidas, exfumador con antecedentes de aneurisma de aorta toraco-abdominal intervenido 2 meses antes con endoprotésis y angioplastia con balón, enfermedad renal estadio 5 por nefroangioesclerosis en diálisis, hipertensión arterial, dislipidemia, hiperparatiroidismo secundario, ferropenia, déficit de ácido fólico. Fue ingresado para colocación de endoprótesis vascular, intervención que se realizó sin incidencias, colocándose endoprótesis ramificada cubriendo ambas arterias renales, arteria mesentérica superior y tronco celiaco, más endoprótesis bifurcada con extensión iliaca derecha e izquierda y en arteria iliaca externa derecha. El paciente permaneció estable tanto hemodinámica como neurológicamente después de la extubación y se trasladó a la unidad de reanimación para control evolutivo. En el postoperatorio inmediato presenta de forma brusca paraparesia recuperada tras drenaje de líquido cefalorraquídeo. El paciente es trasladado a planta de cirugía vascular y 8 días después ingresa nuevamente a la unidad de reanimación por insuficiencia respiratoria aguda asociada a infección respiratoria, que requirió intubación orotraqueal y tratamiento antibiótico con meropenem y linezolid por cultivo de broncoaspirado positivo para h.influenzae. Siendo extubado 2 días después.

En la tarde del día postquirúrgico 13, el paciente presenta melenas, hipotensión arterial y síndrome anémico agudo (hemoglobina 7.4 mg/dl) requiriendo la administración de dos concentrados de hematíes y medidas hemodinámicas farmacológicas; posteriormente refiere aparición de paraparesia con progresión a paraplejía en aproximadamente 8 horas e hipoestesia en ambas extremidades inferiores.

En la exploración neurológica el lenguaje, los pares craneales eran normales. En las extremidades superiores presentaba una fuerza 5/5 sin alteraciones sensitivas con reflejos osteotendinosos conservados. Se evidenció parálisis flácida bilateral de extremidades inferiores, hipoestesia táctil y termoalgésica con nivel T10, apalestesia y areflexia en ambas extremidades inferiores, reflejo cutáneo plantar bilateral indiferente.

Se realizó Angio-TC de Aorta descartándose complicación local en la endoprótesis que pudiera justificar el cuadro clínico y evidenciándose severo derrame pleural izquierdo con atelectasia de lóbulo inferior izquierdo, leve derrame pleural derecho y pequeño infarto en tercio medio posterior esplénico. RM medular mostró imágenes compatibles con infarto agudo de médula dorsal desde aproximadamente el nivel de vértebra D9 a distal, hiperseñal en T2 intramedular en ojos de serpiente, que restringía en la difusión (Figuras 1 y 2).

**Figura Nº 1:**
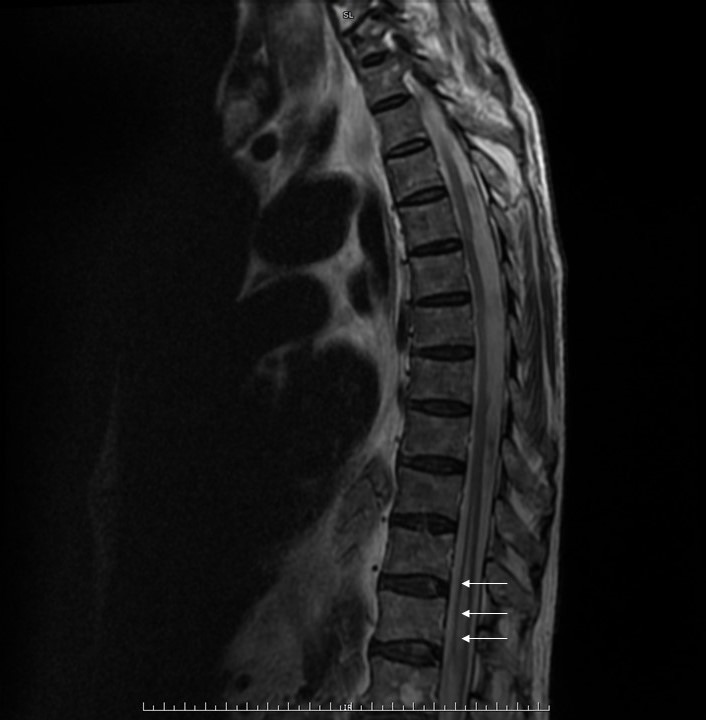
Resonancia magnética de columna dorsal, secuencia T2, corte sagital, en la que se observa hiperintensidad desde D9 "en trazo de lápiz" (flechas blancas).

**Figura Nº 2:**
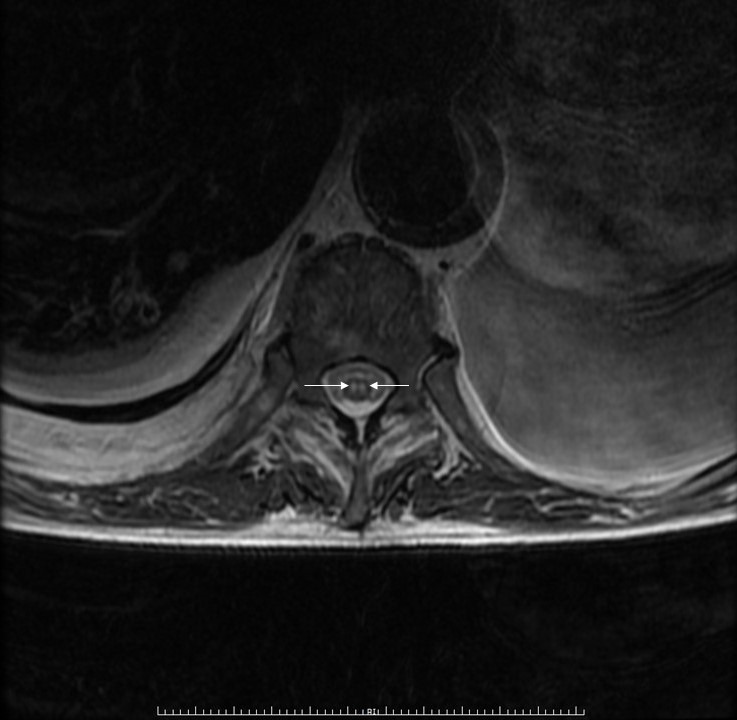
Resonancia magnética de columna dorsal, secuencia T2, corte axial, en la que se observa dos puntos hiperintensos (flechas blancas), "signo de ojos de lechuza".

Debido a que la lesión isquémica ya se encontraba establecida el paciente no fue subsidiario de tratamiento revascularizador. El tratamiento consistió en medidas de soporte, mantener una adecuada perfusión sistémica, vigilancia del estado cardiovascular, respiratorio y neurológico. La situación respiratoria mejoró tras evacuación de derrame pleural mediante colocación de tubo de drenaje torácico y culminar tratamiento antibiótico. Se inició toma de ácido acetilsalicílico y rehabilitación una vez estabilizado el paciente. Al alta el paciente presentaba paraplejia e hipoestesia de ambas extremidades inferiores con incontinencia fecal y urinaria.

## Discusión

El infarto de la médula espinal puede estar limitado a un territorio vascular o estar más extendido según su patogenia (oclusión de una sola arteria o hipoperfusión regional o global). Siendo importante la anatomía vascular para entender la semiología de la isquemia medular. La médula espinal se encuentra irrigada por 3 arterias espinales, una anterior que irriga los dos tercios anteriores de la médula espinal y dos posteriores que irrigan el tercio posterior restante^(6)^, y estás reciben su suministro de sangre de diferentes arterias regionales: C1-T3 es por las arterias vertebrales, T3-T7 por las arterias intercostales, T8 al cono medular por la arteria Adamkiewicz y en algunos casos hay una arteria del cono medular que surge de la arteria ilíaca interna^(2)^. Además, se ha descrito la existencia de una red de colaterales, constituida por pequeñas arterias en el canal espinal, en los tejidos perivertebrales y en los músculos paraespinosos que se anastomosan entre sí y nutren la médula espinal, que incluye también a las arterias subclavia e hipogástrica y sus ramas^(7)^.

La etiología del infarto medular es variada, incluyéndose la cirugía aórtica, la patología aórtica (rotura de aneurisma o disección), la disección de arteria vertebral, la embolia fibrocartilaginosa, los estados de hipercoagulabilidad, el mecanismo cardioembólico o la hipotensión. En un estudio, no se logró determinar la causa en el 29,3% de los pacientes, no obstante, presentaban factores de riesgo vascular^(1)^. Se ha informado que el riesgo de lesión isquémica medular iatrogénica después del injerto aórtico es de hasta 6,5%^(4)^.

Están pocos definidas las causas de lesión medular isquémica relacionadas con el tratamiento endovascular, siendo propuestos como mecanismos fisiopatológicos cobertura de un gran segmento de vasos intercostales y otras colaterales, tales como la subclavia y las arterias hipogástricas; y el robo desde la red colateral vía retrógrada desde los vasos segmentarios hasta los vasos viscerales que comunican con el saco aneurismático. Además, se piensa la hipotensión o trombosis de las arterias intercostales pueden afectar la circulación medular marginal y producir el síndrome de lesión medular tardío^(7)^. En nuestro caso, además de los factores inherentes al tratamiento endovascular, la hipotensión y la anemia secundaria a la rectorragia pudieron afectar el flujo sanguíneo medular produciendo isquemia.

Entre los factores de riesgo asociados a lesiones isquémicas graves de la médula espinal se encuentran: hipertensión arterial, diabetes mellitus y la glucemia elevada al ingreso. En un estudio, la aterosclerosis y la cardioembolia fueron la causa del 14,2 % de todos los infartos espontáneos de la médula espinal^(2)^. El paciente presentado también tenía factores de riesgo vascular que pudieron contribuir al establecimiento del infarto medular.

El inicio de los síntomas es agudo, a menudo caracterizado por dorsalgia localizada generalmente a nivel de la lesión medular, frecuentemente con irradiación radicular. El infarto de la arteria espinal anterior es el más común y se caracteriza por déficits motores bilaterales (principalmente paraparesia) y pérdida de la sensibilidad termoalgésica con mantenimiento de la sensibilidad propioceptiva y vibratoria^(6)^. Puede acompañarse de disfunción autonómica, como hipotensión, disfunción sexual, disfunción intestinal/vesical^(5)^. El infarto de las arterias espinales posteriores es mucho más raro. Se caracteriza por afectación motora bilateral junto con déficit de la sensibilidad vibratoria y propioceptiva^(6)^. Nuestro caso tenía las características clínicas de un infarto de la arteria espinal anterior, sin embargo, también presentaba apalestesia que se encuentra generalmente asociada a afectación de la arteria espinal posterior.

Un signo patognomónico del infarto de la arteria anterior consiste en la conservación de la propiocepción y de la sensibilidad vibratoria por debajo del último segmento sensitivo^(8)^.

En el diagnóstico diferencial se incluye la mielopatía compresiva, enfermedades infecciosas o autoinmunes y otras enfermedades vasculares de la médula como fístulas durales^(1)^.

La RM es muy útil para el diagnóstico de infarto medular. En secuencias potenciadas en T2 la mayor parte de los infartos medulares se manifiestan como lesiones hiperintensas, observándose el llamado patrón "patrón lápiz" En los cortes axiales de las secuencias T2 se puede apreciar el patrón en "ojos de lechuza en aquellos casos donde se encuentra afectada la sustancia gris^(1)^.

En la actualidad no existen guías clínicas sobre el tratamiento óptimo. En relación con el período agudo, hay algunas situaciones aisladas que apoyan el uso de la fibrinólisis intravenosa ^(1)^. Se han realizado mucho esfuerzo con la finalidad de poder predecir y prevenir la isquemia, sin embargo, no se ha conseguir la evidencia necesaria para poder manejar de forma adecuada la lesión isquémica iatrogénica después de que ya se ha presentado ^(4)^. Algunos estudios apoyan el uso de drenaje lumbar en pacientes con infarto medular luego de un procedimiento quirúrgico aórtico. En el resto de los pacientes, se recomienda el control de factores de riesgo vascular y el inicio de antiagregantes o anticoagulantes cuando sea clínicamente apropiado^(1)^, estrategia utilizada en nuestro caso debido a que el infarto ya se encontraba establecido.

La restauración del flujo sanguíneo de la médula espinal podría conducir a una lesión por reperfusión, evento mediado bioquímicamente. El flujo sanguíneo restaurado estimula la expresión de moléculas de adhesión y quimiocinas, que resultaría en un proceso inflamatorio que involucra neurotoxicidad, reclutamiento de leucocitos, daño endotelial de microvasos polimorfonucleares, hipoperfusión y apoptosis^(9)^.

En un estudio realizado con ratas que padecían paraplejía grave inducida por IRI de la médula espinal, se expresaron fuertemente TNF-?, IL-1? y otros mediadores. Algunos investigadores descubrieron que la vía de la proteína quinasa activada por mitógeno/quinasa regulada por señal extracelular (MEK/ERK) podría desempeñar un papel nocivo al participar en reacciones inflamatorias y la producción de citoquinas^(9)^.

Además, la interrupción de la barrera hematomedular es un cambio patológico importante que puede exacerbar el edema de la médula espinal, aumentar la infiltración de leucocitos y amplificar la inflamación y el estrés oxidativo. Por lo tanto, la interrupción de la barrera hematomedular juega un papel vital en la evolución de la lesión medular por isquemia-reperfusión y el daño adicional de las neuronas^(10)^.

Algunas técnicas han demostrado disminuir la presentación de daño medular, algunas de ellas son: técnicas de perfusión distal, hipotermia, prevención del fenómeno de robo por la red de colaterales, drenaje de líquido cefalorraquídeo y maximización de los parámetros de perfusión^(7)^.

Con respecto al pronóstico, los pacientes con infarto de la médula espinal mayormente asociados a cirugía, aneurisma o disección aórtica presentan a largo plazo mayor mortalidad, llegando a un 23% luego de 3 años de seguimiento. Dos tercios de los pacientes con infarto medular pueden caminar durante el seguimiento^(2)^.

Concluyendo, el infarto medular es una patología de baja frecuencia, cuyas manifestaciones clínicas aparecen de forma brusca, pudiendo llegar a producir un gran impacto en la calidad de vida de los pacientes. La exploración neurológica es muy importante en el período postoperatorio debiendo mantener un alto grado de sospecha para identificar causas tratables. En aquellos pacientes con clínica compatible es necesaria la realización de una RM, que podría mostrar lesiones hiperintensas en T2. Sin embargo, se necesitan más estudios para dilucidar los mecanismos que subyacen a las lesiones medulares y evaluar mejores estrategias de prevención, así como de tratamiento.
